# Find Outliers of Image Edge Consistency by Weighted Local Linear Regression with Equality Constraints †

**DOI:** 10.3390/s21072563

**Published:** 2021-04-06

**Authors:** Mingzhu Zhu, Yaoqing Hu, Junzhi Yu, Bingwei He, Jiantao Liu

**Affiliations:** 1 BIC-ESAT, Department of Advanced Manufacturing and Robotics, College of Engineering, Peking University, Beijing 100089, China; mzz@pku.edu.cn; 2 School of Automation and Electrical Engineering, University of Science and Technology Beijing, Beijing 100089, China; S20180573@xs.ustb.edu.cn; 3 Institute of Mechanical Engineering, Fuzhou University, Fuzhou 350000, China; mebwhe@fzu.edu.cn (B.H.); liujiantao@gmail.com (J.L.)

**Keywords:** outlier detection, edge consistency, post-refinement, weight map

## Abstract

In this paper, we propose a general method to detect outliers from contaminated estimates of various image estimation applications. The method does not require any prior knowledge about the purpose, theory or hardware of the application but simply relies on the law of edge consistency between sources and estimates. The method is termed as ALRe (anchored linear residual) because it is based on the residual of weighted local linear regression with an equality constraint exerted on the measured pixel. Given a pair of source and contaminated estimate, ALRe offers per-pixel outlier likelihoods, which can be used to compose the data weights of post-refinement algorithms, improving the quality of refined estimate. ALRe has the features of asymmetry, no false positive and linear complexity. Its effectiveness is verified on four applications, four post-refinement algorithms and three datasets. It demonstrates that, with the help of ALRe, refined estimates are better in the aspects of both quality and edge consistency. The results are even comparable to model-based and hardware-based methods. Accuracy comparison on synthetic images shows that ALRe could detect outliers reliably. It is as effective as the mainstream weighted median filter at spike detection and is significantly better at bad region detection.

## 1. Introduction

Edge consistency is crucial in many image processing applications producing per-pixel estimates based on source images, such as depth estimation, object segmentation and alpha matting. Although these applications have very different models and purposes, the estimates are all expected to have consistent edges with the sources.

Edge consistency has a popular description known as “output image has an edge only if input image has an edge”.The meaning of this description is twofold. Firstly, it means “estimate should be smooth if the source is smooth”.Secondly, it means “estimate could be smooth whether the source has an edge or not”.It is reasonable since most estimation processes are not single mappings. Pixels with different values might be the same after mapping. Therefore, the source and estimate have different status in the context of edge consistency.

Although the estimates are expected to fulfill edge consistency, it is not usually the case due to noises and outliers. Various algorithms have been proposed for noise suppression including global optimizations [1,2,3] and local filters [4,5]; however, outliers are less handled in the context of images [6,7,8,9].

Noises are derived from sensors and environments, leading to small biases which can usually be well described by statistical models. Outliers are caused by unexpected samples or improper designs of processing methods (termed artifacts in this situation). They might trigger large offsets and have very different forms between applications. Therefore, outliers are hard to model and suppress in a general way. Options on the table are limited:

1. Adopt median filter;
2. Ignore and treat them as noises;
3. Design prior/model/hardware based methods;
4. Train a data-driven method [10].

Median filter and its improved versions are good at removing small outlier regions such as spikes [11,12,13]. However, outliers might occupy large regions and happen to be the medians. Noise suppression methods are usually not aggressive enough to remove outliers, leaving artifacts such as halo-effect. In some applications, outlier detection methods are specifically designed based on prior, model or hardware. These methods are effective but not general. In most cases, we do not have a satisfactory option.

In this paper, we show that edge consistency itself is a valuable and under-exploited clue for general outlier detection. A hypothesize-and-verify algorithm termed ALRe (anchored linear residual) is proposed to find pixels undermining edge consistency. It offers per-pixel outlier likelihoods of estimates based on the source. The likelihoods can be transformed into inlier fidelities and then used as data weights of various post-refinement algorithms, producing better refined estimates. The algorithm requires no prior knowledge about applications or estimation method and has the complexity linear to pixel number. To the best of our knowledge, ALRe is the first general outlier detection method based on edge consistency. A preliminary version of ALRe is published in ECCV 2020 [14]. In this paper, we provide the full feature analysis and quantitative results on various applications, and compare its detection accuracy with the mainstream weighted median filter.

The rest of this paper is organized as follows. Section 2 introduces post-refinement algorithms and several limited outlier detection methods. Section 3 presents the details of ALRe. Section 4 analyzes its advantages and compares some other options. Section 5 gives examples. In Section 6, the value of ALRe as an add-on of post-refinements is investigated. Furthermore, its accuracy is tested on synthetic images. Conclusion is given in Section 7.

## 2. Related Works

Although corresponding outlier detection method is absent, edge consistency has been considered in various algorithms, such as WLS (weighted least squares) [2], JBF (joint bilateral filter) [4], GF (guided filter) [5] and WMF (weighted median filter) [11]. Note that these algorithms have multiple usages. The following introduction only includes post-refinement for image estimation. In this situation, they pursue a refined estimate *q*, which has similar intensities with the contaminated estimate *p* and consistent edges with the source I.

### 2.1. Weighted Least Squares

WLS [2] has a straightforward definition following the concept of edge consistency closely. It finds the optimal *q* minimizing the energy function

(1) $$E\left(q\right)=\sum_{i}\underset{▵}{w_{i}}{\left(q_{i}-p_{i}\right)}^{2}+\lambda \sum_{(i,j)\in J}a_{ij}{\left(q_{i}-q_{j}\right)}^{2},$$

where *i* is pixel index, *J* is the set of adjacent pixels, and λ balances the two terms. Smooth weight $a_{ij}$ is inversely correlated with the distance between $I_{i}$ and $I_{j}$. WLS are continuously improved, such as efficient semi-global WLS [15] and constrained WLS [16], to keep pace with its applications. However, the data weight $w_{i}$ correlated with the fidelity of $p_{i}$ is usually undefined.

### 2.2. Joint Bilateral Filter

JBF [4] produces *q* by smoothing *p* based on I, as

(2) $$\left\{\begin{array}{cc} q_{i} & =\sum_{j\in \Omega_{i}}K_{ij}p_{j}\\ K_{ij} & =\frac{1}{Z_{i}}\underset{▵}{w_{i}}s_{ij}c_{ij}\\ s_{ij} & =exp\left(-\frac{\left|\right|x_{i}-x_{j}{\left|\right|}^{2}}{\sigma_{s}^{2}}\right)\\ c_{ij} & =exp\left(-\frac{\left|\right|I_{i}-I_{j}{\left|\right|}^{2}}{\sigma_{c}^{2}}\right) \end{array}\right.,$$

where x is the vector of pixel coordinate, $Z_{i}$ is normalizing parameter, and $\Omega_{i}$ is the local region centered at pixel *i*. There are two kinds of weights including distance weight *s* and color weight *c*. The parameters $\sigma_{s}$ and $\sigma_{c}$ adjust the sensitivities of the spatial and color similarities, respectively. Bilateral filter have many other variants with similar structures, such as guided bilateral filter [17] and optimally weighted bilateral filter [18]. However, the data weight $w_{j}$ is usually undefined.

### 2.3. Guided Filter

GF [5] assumes local linear relationship between *q* and I and then solves the optimal *q* closest to *p*. It is defined as

(3) $$\left\{\begin{array}{cc} q_{i} & =\frac{1}{\left|\Omega \right|}\sum_{k\in \Omega_{i}}w_{ik}^{a}\left(a_{k}^{T}I_{i}+b_{k}\right)\\ \left(a_{k},b_{k}\right) & =\arg\min_{a_{k},b_{k}}\sum_{j\in \Omega_{k}}\left(R_{jk}\left(a_{k},b_{k}\right)+\epsilon w_{k}^{w}a_{k}^{T}a_{k}\right)\\ R_{jk}\left(a_{k},b_{k}\right) & =\underset{▵}{w_{j}}w_{jk}^{t}{\left(a_{k}^{T}I_{j}+b_{k}-p_{j}\right)}^{2} \end{array}\right.,$$

where a and *b* are linear parameters, ϵ suppresses large a for smoothness, and |Ω| means the pixel number of Ω. GF has been improved into many versions. Anisotropic guided filter [19] contains the weight $w_{ik}^{a}$, and weighted guided filter [20] contains $w_{k}^{w}$. Dai et al. [21] relaxed local support region Ω to the entire image domain and introduces the weight $w_{jk}^{t}$ based on minimum spanning tree. Additionally, a kind of bidirectional guided filter can be found in [22]. These methods introduce various benefits, such as stronger edge-preserving behavior and less halo-effect. However, to take advantage of pixel fidelities, another kind of weight $w_{j}$ is required. It is not originally included in [5] but can be easily implemented without increasing complexity.

### 2.4. Weighted Median Filter

WMF [11] produces *q* by picking values from *p*. It is robust to outliers because unpicked pixels have no impact on the result. WMF is defined as

(4) $$\left\{\begin{array}{cc} h(i,v) & =\sum_{j\in \Omega_{i}}\underset{▵}{w_{j}}w_{i,j}\delta \left(p_{j}-v\right)\\ q_{i} & =v^{\prime }\\ \sum_{v=l}^{v^{\prime }}h(i,v) & ⩽\frac{1}{2}\sum_{v=l}^{u}h(i,v)\\ \sum_{v=l}^{v^{\prime }+1}h(i,v) & >\frac{1}{2}\sum_{v=l}^{u}h(i,v) \end{array}\right.,$$

where δ(x) is 1 if *x* equals 0, and is 0 otherwise. The weight $w_{i,j}$ depends on $I_{i}$ and $I_{j}$ (in this paper, it is produced based on the kernel of guided filter). Median filter has been improved in both robustness [12] and efficiency [13]. However, they might fail when filter size is large or some outliers happen to be the medians. This problem can be improved if the fidelity of each single pixel is available. It requires the weight denoted as $w_{j}$. It is not originally included in [11] but can be easily implemented without increasing complexity.

### 2.5. Outlier Detection

In the field of haze removal, Fattal [23] proposed color-line model and Berman et al. [24] proposed haze-line model. Outliers in their initial estimates are detected based on the variances of fitted color-lines and the effective lengths of collected haze-lines, respectively. WLS is employed for post-refinement, and the data weight *w* is provided based on the detection results. It introduces robustness since pixels not following their models affect the final estimates little. However, these detection methods are only applicable to corresponding models and thus cannot be generalized to other models and applications.

In the field of disparity estimation, outliers can be robustly detected by cross check [25]. Pixels having different estimates between left-to-right and right-to-left matchings are considered as outliers, and their weights are set to zeros. JBF is employed for post-refinement, whose data weight *w* is provided based on the detection results. Despite the robustness, cross-check is also not generalizable because it requires multiple source images captured by specific hardware.

More often, outlier detection method is absent, and the post-refinement algorithms are used without data weights. In this case, outliers are treated as noises and not well removed. However, most of them are obviously in the view of edge consistency. In this paper, we propose ALRe to realize this simple, general and effective check.

## 3. Method

### 3.1. Intuition

ALRe is based on the fact that estimate *p* and source I satisfy edge consistency if local linear relationship is established between them. Denote the local region centered at *k* by $\Omega_{k}$; the local linear assumption is satisfied if

(5) $$p_{i}=a_{k}^{T}I_{i}+b_{k},\;i\in \Omega_{k},$$

where $\left(a_{k},b_{k}\right)$ are linear parameters. It implies edge consistency because

(6) $$\Delta p_{i}=a_{k}\Delta I_{i}.$$

On the other hand, edge consistency does not always imply local linear relationship, especially when $I_{\Omega_{k}}$ has notably more edges than $p_{\Omega_{k}}$. However, with proper mask size, $\Omega_{k}$ contains few edges and colors; thus, the assumption is reasonable enough in most cases.

Linear regression can be used in each $\Omega_{k}$ to approach Equation (5) as much as possible. Based on the relationship between local linear assumption and edge consistency, the smaller the residual, the better the consistency. However, simply adopting linear regression has two problems:

1. The residual of linear regression indicates the degree of edge consistency in $\Omega_{k}$, rather than the fidelity of the single pixel *k*. It can not help post-refinement algorithms;
2. All the pixels in $\Omega_{k}$ are considered as inliers. Outliers have strong impacts to the regression, especially when the least square regression is used.

To solve these problems, we refer to RANSAC (random sample consensus) [26], which is a hypothesize-and-verify algorithm that firstly assumes inliers and then calculates the fidelity of the inlier assumption based on all the samples. We firstly make inlier assumption at each pixel *k* and then evaluate the assumption in $\Omega_{k}$. For each pixel *k*, we

1. Assume edge consistency in $\Omega_{k}$;
2. Assume $p_{k}$ is an inlier;
3. Evaluate the two assumptions by weighted regression;
4. Calculate the inlier fidelity of $p_{k}$ based on the residual.

When it is complete, a fidelity map that has the same size as *p* is available. It is transformed into data weights *w* and then used in the next round regressions to further suppress the impacts of outliers.

### 3.2. Algorithm

The edge consistency assumption and the inlier assumption imply a small residual *e* defined as

(7) $$\left\{\begin{array}{cc} e_{k} & =\min_{a_{k},b_{k}}\frac{1}{\sum_{i\in \Omega_{k}}w_{i}}\sum_{i\in \Omega_{k}}w_{i}{\left(a_{k}^{T}I_{i}+b_{k}-p_{i}\right)}^{2}\\ p_{k} & =a_{k}^{T}I_{k}+b_{k} \end{array}\right..$$

The inlier fidelity *w* is negatively correlated with *e* as

(8) $$w_{k}=\frac{\frac{1}{\max(\mathrm{LB},\min\left(\mathrm{UB},\sqrt{e_{k}}\right))}-\frac{1}{\mathrm{UB}}+\epsilon }{\frac{1}{\mathrm{LB}}-\frac{1}{\mathrm{UB}}+\epsilon },$$

where (LB,UB) are the lower bound and upper bounds of $\sqrt{e}$. When $e_{k}$ is out of the bounds, pixel *k* is considered as absolute inlier and outlier, respectively. ϵ is a small number for numerical stability.

The energy function and the equality constraint of Equation (7) can be combined into

(9) $$e_{k}=\min_{a_{k}}\frac{\sum_{i\in \Omega_{k}}w_{i}{\left(a_{k}^{T}\left(I_{i}-I_{k}\right)-\left(p_{i}-p_{k}\right)\right)}^{2}}{\sum_{i\in \Omega_{k}}w_{i}}.$$

By further induction, we have

(10) $$\left\{\begin{array}{cc} C_{k} & =\sum_{i\in \Omega_{k}}w_{i}\left(I_{i}-I_{k}\right){\left(I_{i}-I_{k}\right)}^{T}\\ d_{k} & =\sum_{i\in \Omega_{k}}w_{i}\left(p_{i}-p_{k}\right)\left(I_{i}-I_{k}\right)\\ a_{k} & =C_{k}^{-1}d_{k}\\ b_{k} & =p_{k}-a_{k}^{T}I_{k} \end{array}\right..$$

In programming, it is

(11) $$\left\{\begin{array}{cc} C_{k} & ={\left(\bar{wII^{T}}\right)}_{k}+{\left(\bar{w}\right)}_{k}I_{k}I_{k}^{T}-{\left(\bar{wI}\right)}_{k}I_{k}^{T}-I_{k}{\left(\bar{wI^{T}}\right)}_{k}+\epsilon \\ d_{k} & ={\left(\bar{wpI}\right)}_{k}-p_{k}{\left(\bar{wI}\right)}_{k}-{\left(\bar{wp}\right)}_{k}I_{k}+{\left(\bar{w}\right)}_{k}p_{k}I_{k}\\ a_{k} & =C_{k}^{-1}d_{k}\\ b_{k} & =p_{k}-a_{k}^{T}I_{k}\\ e_{k} & =(a_{k}^{T}{\left(\bar{wII^{T}}\right)}_{k}a_{k}+{\left(\bar{w}\right)}_{k}b_{k}^{2}+{\left(\bar{wp^{2}}\right)}_{k}+2b_{k}a_{k}^{T}{\left(\bar{wI}\right)}_{k}\\ \; & -2a_{k}^{T}{\left(\bar{wpI}\right)}_{k}-2b_{k}{\left(\bar{wp}\right)}_{k})/\left({\left(\bar{w}\right)}_{k}+\epsilon \right) \end{array}\right.,$$

where ϵ is a diagonal matrix whose elements all equal ϵ, ${\left(\bar{p}\right)}_{k}$ is the mean value of *p* in $\Omega_{k}$, and so do the others.

Since *e* and *w* are interdependent, an iteration strategy with $w^{0}=1$ is employed as

(12) $$\begin{array}{c} w^{0}\to \ldots \to w^{t}\to e^{t+1}\to w^{t+1}\to \ldots , \end{array}$$

and the terminal condition is

(13) $$\Delta e^{t+1}=\sum_{k}|e_{k}^{t+1}-e_{k}^{t}|<\epsilon .$$

In practice, the iteration number is usually 5∼10.

The overall algorithm is summarized in Algorithm 1. The final *w* is inlier fidelity and 1−w is outlier likelihood. Note that, those mean values can be calculated by boxfilter with O(N) complexity, where *N* is the pixel number. Other operations in Equation (8) and Equation (11) are O(N) too. The number of iterations is independent of *N*. Therefore, the algorithm is O(N) overall. Compared to linear regression without equality constraint [5], which leads to a solution similar to Equation (10), only several mean values are replaced by particular ones. The runtime is even slightly decreased. When handling 640×480 images, each iteration of our ALRe demon (provided in https://github.com/Lilin2015/Author—ALRe (accessed on 5 April 2021)) takes about 0.3 s.
**Algorithm 1** ALRe**Require:** I,p,ϵ,LB,UB**Ensure:** w 1:set $t=0,\Delta e^{t}=1$ 2:set $e^{t}=1,w^{t}=1$ for all *k* 3:**while**$\Delta e^{t}⩾\epsilon$**do** 4:  calculate $\bar{w^{t}},\bar{w^{t}p},\bar{w^{t}I},\bar{w^{t}p^{2}},\bar{w^{t}II^{T}},\bar{w^{t}pI}$ by boxfilter 5:  **for** each *k* **do** 6:   calculate $e_{k}^{t+1}$ by Equation (11) using $w_{k}=w_{k}^{t}$ 7:   calculate $w_{k}^{t+1}$ by Equation (8) using $e_{k}=e_{k}^{t+1}$ 8:  **end for** 9:  $\Delta e^{t+1}=\sum_{k}|e_{k}^{t+1}-e_{k}^{t}|$ 10:  t=t+1 11:**end while** 12:$w=w^{t}$

## 4. Analysis

### 4.1. Asymmetry

Linear transformations on I and shifts on *p* have no impact on the result of *w*. Firstly, with fixed *w*, the conclusion of

(14) $$e\left(p+\beta_{p},\alpha_{I}I+\beta_{I}\right)=e\left(p,I\right)$$

can be proven based on Equation (10) as

(15) $$\left\{\begin{array}{cc} \tilde{I} & =\alpha_{I}^{T}I+\beta_{I}\\ \tilde{p} & =p+\beta_{p} \end{array}\right.\Rightarrow \left\{\begin{array}{cc} \tilde{C} & =\alpha_{I}^{2}C\\ \tilde{d} & =\alpha_{I}d \end{array}\right.\Rightarrow \left\{\begin{array}{cc} \tilde{a} & =\frac{a}{\alpha_{I}}\\ \tilde{b} & =b \end{array}\right.\Rightarrow \tilde{e}=e.$$

Then, the same *e* results in the same *w*.

On the other hand, with fixed *w*, a scaling on *p* leads to

(16) $$e\left(\alpha_{p}p,I\right)=\alpha_{p}^{2}e\left(p,I\right).$$

If we simply consider the inverse relationship between $\sqrt{e}$ and *w*, it results in $\tilde{w}=w/\alpha_{p}$, but the real situation is more complex due to the truncated mapping. Anyway, it is clear that ALRe has asymmetric responds to I and *p*.

The asymmetry fulfills the concept of edge consistency. Contemplate a pair of I and *p* with modest sharpness

1. When $\alpha_{p}$ is large, *p* has sharp edges, *e* is small only if I and *p* closely follow the local linear assumption because of the large factor $a_{p}^{2}$ in Equation (16). It corresponds to the description “estimate should be smooth if the source is smooth”;
2. When $\alpha_{p}$ is small, *p* is smooth, *e* is small because of the small $a_{p}^{2}$. The sharpness of I is unessential. It corresponds to the description “estimates could be smooth whether the source has an edge or not”.

In most applications, I has much more edges than *p*, but it does not always lead to small *w* because of this asymmetry.

### 4.2. No False Positive

The main difference between each iteration of ALRe and LRe (linear residual, produced by naive least square regression) is the equality constraint. Previous discussion shows that introducing this constraint does not cost the feature of asymmetry. However, what is the benefit?

To answer this question, we conduct simulations. Firstly, random I and (a,b) are generated in [0,1]. Then, *p* is calculated by $p_{k}=a_{k}^{T}I_{k}+b_{k}$. Based on a preset bad pixel ratio, some pixels of *p* are modified by random biases in [0.05,0.5]. Ten levels of bad pixel ratio, from 5% to 50%, are tested. For each level, $10^{5}$ sources and contaminated estimates are generated. Linear residuals of bad pixels with and without equality constraint are recorded.

**Figure 1:**
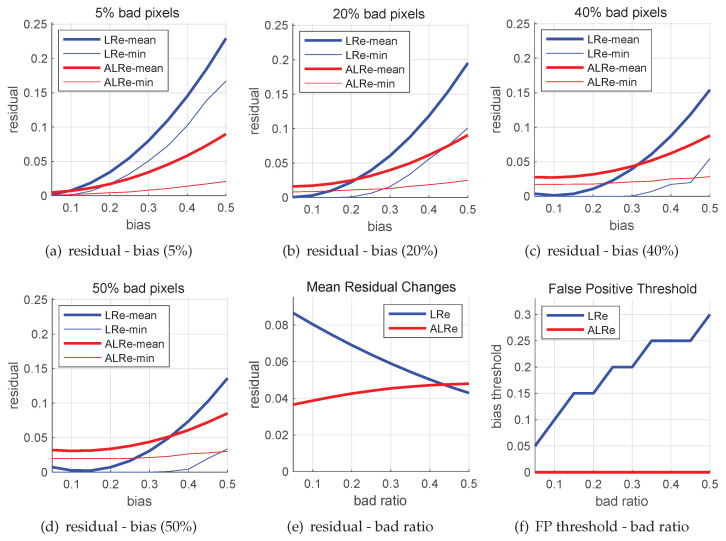
Bad pixel residuals of linear regression with and without equality constraint. Marked as ALRe and LRe, respectively. (**a**–**d**) Mean and minimal residuals of bad pixels with different biases; (**e**) Mean residuals of bad pixels under different configurations of bad pixel ratio; (**f**) Under different bad pixel ratios, the thresholds that pixels with smaller biases might have zero residuals.

The results are shown in Figure 1, where LRe means the naive linear residual, and ALRe means the anchored linear residual of the first iteration.

The results of four levels of bad pixel ratio are displayed in Figure 1a–d respectively. As expected, both LRe and ALRe increase when the bias increases. LRe is more sensitive to large biases and ALRe is more sensitive to small biases. However, as the bad pixel ratio increases, mean LRe decreases while mean ALRe keeps stable or even slightly increases. Figure 1e illustrates this phenomenon, where the slope of ALRe is obviously smaller. It means that ALRe is more robust to bad pixel ratio. This is the first benefit.

More importantly, if we further investigate the minimal residual of each test, it comes out that LRe has a false positive problem. The minimal LRe and ALRe are drawn as thin curves in Figure 1a–d. As can be seen, some segments of the blue curves are overlapped with x-axis when the biases are small. The larger the bad pixel ratio, the longer the overlapped segment. For convenience, we term the x-value of the right end of the overlapped segment as false positive threshold, since bad pixels with biases smaller than this value might have zero residuals and be recognized as inliers. Figure 1f illustrates this phenomenon, where the false positive threshold of LRe increases when the bad pixel ratio increases. As a comparison, ALRe has no false positive problem. This is the second and the major benefit.

## 5. Applications

We provide four examples of using ALRe, including haze removal, depth estimation, feathering and edge-preserving smoothing. ALRe affects these applications by providing or replacing the data weights in their post-refinements.

### 5.1. Transmission Refinement for Haze Removal

In the field of haze removal, hazy images are considered as haze-free images attenuated by atmospheric lights and transmission maps represents the attenuation ratios. With evenly dispersed haze, attenuation ratios are related to scene depths. Therefore, transmission edges should be consistent with depth edges. Since depth edges are unavailable and mostly happen on color edges, transmission maps are expected to have edge consistency with hazy images.

**Figure 2:**
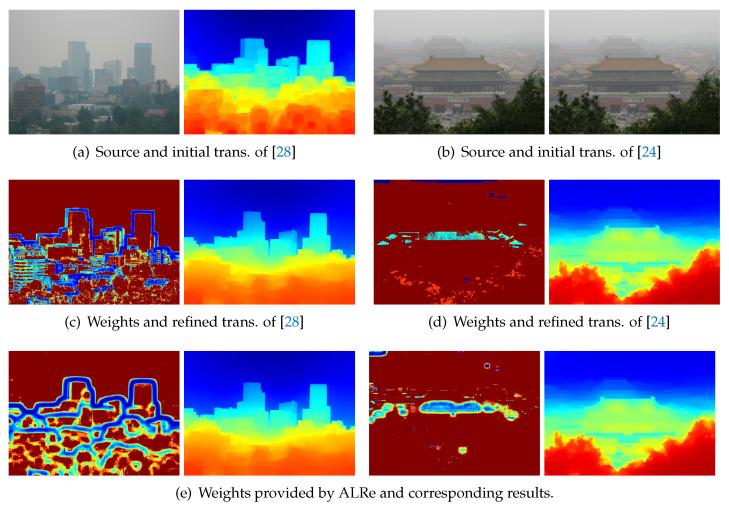
Transmission map refinements. Transmission and weight maps are displayed in color. The warmer the color, the larger the value. (**a**,**b**) Hazy images and the initial transmission maps based on dark channel prior [27] and haze-line model [24] respectively; (**c**,**d**) The weight maps and the refined results based on Zhu et al. [28] and Berman et al. [24] respectively; (**e**) The weight maps based on ALRe and corresponding results.

However, limited by existing technologies, transmission maps usually have unsatisfactory edge consistency; thus a post-refinement based on hazy images is popular. Two examples based on dark channel prior [27] and haze-line model [24] are illustrated in Figure 2. Initial transmission map based on dark channel prior has the problem of block effect, which indicates over-estimated transmissions in the vicinity of large depth jumps [27]. As displayed in Figure 2a, edges of the two images are misaligned near the skyline, where large transmission values of the distant buildings are dilated. Initial transmission map based on haze-line model is estimated using the assumption that the end of each haze-line is haze-free [24]. The assumption is unreliable for short haze-lines, introducing isolated outliers, such as the over-estimated pixels on the sky and roof.

To address the problem of block effect, Zhu et al. [28] detect outliers based on an improved local constant assumption customized for dark channel prior and then employs WLS. As shown in Figure 2c, with correctly revealed outliers, the block effect is well removed without introducing halo-effect. Since the initial transmission map based on haze-line model is unreliable on short haze-lines, Berman et al. [24] detect outliers based on the effective length of haze-line and then employ WLS. As shown in Figure 2d, outliers are correctly revealed, and the transmission map is well refined.

Despite the effectiveness, these outlier detection methods are based on the deep understanding of the assumption, prior and model applied in estimation process. As a comparison, ALRe has similar performance without any of these knowledges. As shown in Figure 2e, according to the law of edge consistency, outliers are also revealed. The refined results based on WLS have trivial differences compared to Zhu et al. [28] and Berman et al. [24].

### 5.2. Refinements in Depth Estimation

**Figure 3:**
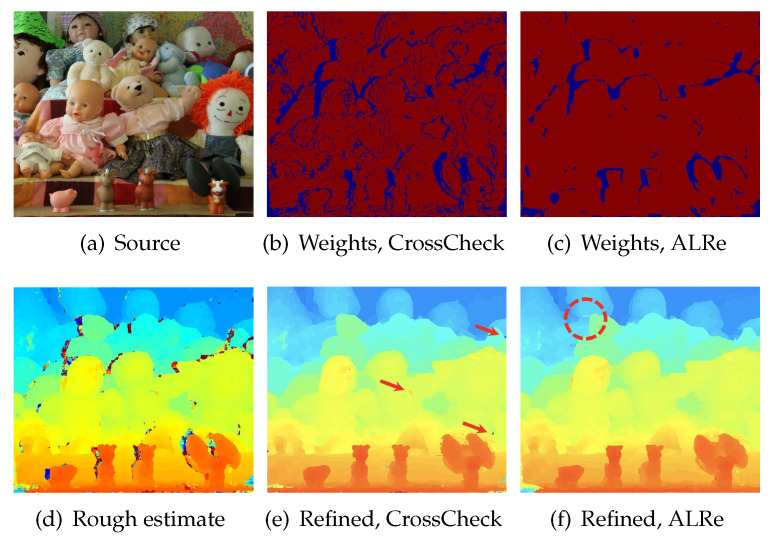
Disparity map refinements. Disparity and weight maps are displayed in color. The warmer the color, the larger the value. (**a**) One of the two stereo images; (**b**) the weight map based on cross check; (**c**) the weight map based on ALRe (the segmentation threshold is 0.2); (**d**) initial disparity map based on Hosni et al. [25]; (**e**) the refined map based on (**b**); (**f**) the refined map based on (**c**).

Disparity refers to the difference in image locations of a point seen from different views. Disparity maps are inversely proportional to depth maps, whose edges are consistent with color edges. Therefore, they are also expected to have edge consistency with color images. Disparities can be estimated by stereo matching. However, it might be false or invalid on several pixels due to occlusions. An example from Middlebury dataset 2005 [29,30] is shown in Figure 3a (one of a pair). The initial disparity map in Figure 3d is generated by Hosni et al. [25] (without refinement). Outliers can be seen on the left side of most dolls.

Hosni et al. [25] traced outliers by cross check, which requires the image of another view. ALRe is applicable without this extra information. The binary results of these two methods are shown in Figure 3b,c, and the refined maps are displayed in Figure 3e,f. As can be seen, although the seams are missed, ALRe reveals most occluded regions. The refined results are similar and comparable even though ALRe has much less information.

**Figure 4:**
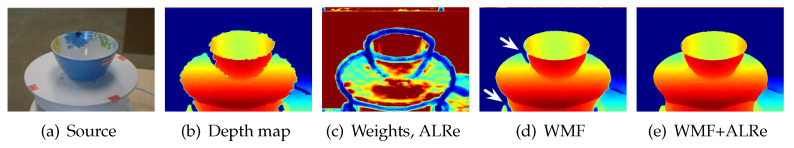
Depth map refinements. Depth and weight maps are displayed in color. The warmer the color, the larger the value. (**a**) Color image; (**b**) rough depth map; (**c**) the weight map based on ALRe; (**d**) the refined result based on WMF without ALRe; (**e**) corresponding result with ALRe.

Cross check is not always valid because it requires at least two views of the same object. It is invalid to single view equipment such as RGB-D camera. The depth map of RGB-D camera usually contains unstable edges due to the shifted position and low resolution of depth camera, as illustrated in Figure 4a,b. This problem can be solved by WMF. As shown in Figure 4d, the winding edges are well regularized, but the values of the pointed regions are wrong picked. These regions have zero values because they are invisible to the depth camera and thus should not be considered in picking. With the help of ALRe, these regions are trivial in WMF, and a more convincing result is achieved.

### 5.3. Feathering

**Figure 5:**
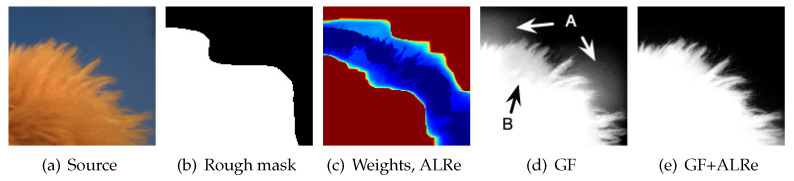
Feathering. Weight map is displayed in color. The warmer the color, the larger the value. (**a**) Input image; (**b**) rough mask; (**c**) the weight map based on ALRe; (**d**) the refined result based on GF without ALRe; (**e**) corresponding result with ALRe.

Image feathering produces an alpha matte of complex object based on rough binary mask, which can be obtained manually or from other segmentation methods. GF is an efficient tool but not error-tolerant enough since it treats all pixels equally. Masks with obvious errors might lead to halo-effects. As shown in Figure 5d, the result of GF inherits the errors from Figure 5b, leading to the over-estimated and under-estimated results marked by A and B, respectively.

As displayed in Figure 5c, the weights of the mask are very low near the boundaries. It is not surprising since rough masks are never expected to have consistent edges with color images. However, with a closer look, phantom of the edges from both images can be observed, and the regions between them have almost zero fidelities. With this message, a more convincing matte is produced as shown in Figure 5e.

### 5.4. Edge-Preserving Smoothing

Edge-preserving smoothing aims to erase weak edges but preserve strong ones from images. As an edge-preserving filter, GF has the problem of halo-effects. Various methods have been proposed for this problem and various weights have been investigated as described in Equation (3).

**Figure 6:**
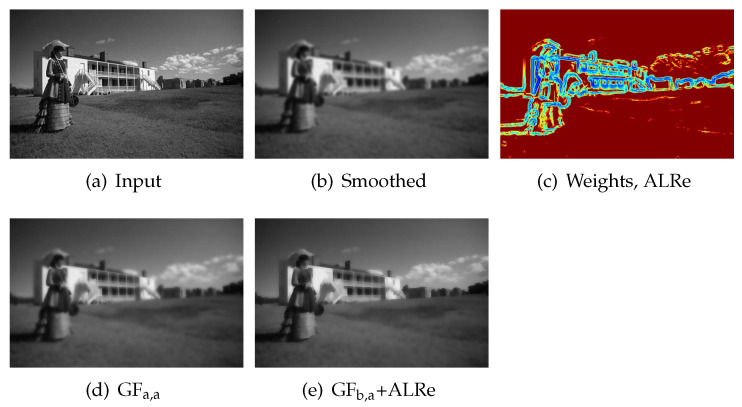
Edge preserving filtering. Weight map is displayed in color. The warmer the color, the larger the value. (**a**) Input image; (**b**) the smoothed input image based on Gaussian low-pass filter; (**c**) the weight map of (**b**) based on ALRe; (**d**) the smoothed result of (**a**) guided by itself based on GF; (**e**) the enhanced result of (**b**) guided by (**a**) based on GF with ALRe.

ALRe also has the potential to discover strong edges. Firstly, we smooth sharp input by low-pass filter, as shown in Figure 6b. After this indiscriminatingly suppression, weak edges are almost erased but strong edges are still observable. Then, we investigate the edge consistency between the sharp input and the smoothed result. Stronger edges contribute larger residuals, as described in Equation (16) and confirmed in Figure 6c and thus can be recognized and enhanced back.

Therefore, with the help of ALRe, edge-preserving can be achieved by enhancing smoothed result guided by sharp input. In this process, GF copies strong edges back in low weight regions. As shown in Figure 6e, the lawn is as smooth as the one in Figure 6d, but the halo-effects near the skyline are avoided.

### 5.5. Summary

Different from noises and biases, outliers are from theoretical or system flaws. They are usually far away from true results and severe against the law of edge consistency. As shown in the examples above, outliers introduce different estimates on the same object, large value jump between similar colors or unexpected edges on a flat region. Most of them can be found without any knowledge but simply the expectation of edge consistency. Compared to prior-based, model-based and hardware-based outlier detection methods, ALRe is unlikely to be the best. However, as a general method, ALRe can be used when specific method is absent, which is a considerable situation. Furthermore, it can be used an extra add-on of existing methods.

## 6. Experiments

In this section, we conduct two kinds of quantitative experiments. Firstly, the value of ALRe as an add-on of transmission refinement, disparity refinement and feathering is investigated. Secondly, its outlier detection accuracy is measured and compared with WMF (the kernel of guided filter is adopted) based on synthetic images.

In the first part, D-HAZY [31], Middlebury [32] and Alpha Matting [33] datasets are employed (They are public datasets. The permissions can be found on the dataset websites. D-HAZY: http://ancuti.meo.etc.upt.ro/D_Hazzy_ICIP2016/ (accessed on 5 April 2021); Middlebury: https://vision.middlebury.edu/stereo/data/ (accessed on 5 April 2021); Alpha Matting: http://www.alphamatting.com/index.html (accessed on 5 April 2021)).Comparisons are made based on rough outputs, refined outputs without ALRe and refined outputs with ALRe. The index used to measure edge consistency is ESR (mean edge strength ratio).

(17) $$\mathrm{ESR}\left(q,I\right)=\frac{1}{N}\sum_{k\in q}\frac{|G(q_{k})|+\epsilon }{|G(I_{k})|+\epsilon }.$$

ESR exactly follows the concept of edge consistency but lacks a reasonable minimum. For example, results with constant values always have minimal ESR. Therefore, to make sure what ALRe introduces are improvements rather than over-smoothing, additional metrics measuring output qualities are required. In both parts, the ϵ, LB and UB are 0.001, 0.01 and 0.3, respectively. The size of Ω depends.

### 6.1. Transmission Refinement

D-HAZY [31] is a popular dataset which provides abundant hazy and haze-free image pairs. It is built based on Middlebury [32] and NYU Depth [34] respectively. In this comparison, we only use the Middlebury part because of the significantly higher quality. The MSE (mean square error) between haze removal result and groundtruth is used for quality measurement.

In practice, we found out that global biases dominate the results of Berman et al. [24], while outliers are trivial. As a comparison, the outliers of block effect in the rough outputs of Zhu et al. [28] challenge both post-refinement and ALRe. Therefore, the experiment is based on Zhu et al. [28]. We exactly follow the configurations of Zhu et al. [28], and the Ω for ALRe has the same size (51×51). For each sample, we produce the rough transmission map with block effect, the refined map based on WLS without ALRe and the refined map with ALRe. Transmission maps are evaluated by ESR, and haze removal results are evaluated by MSE.

**Figure 7:**
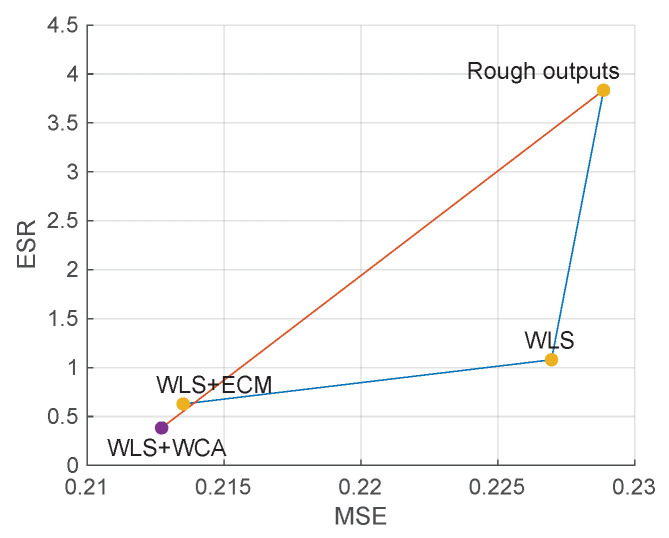
Effect of ALRe on WLS in transmission refinement. The ends of the orange line are the mean errors of rough and refined outputs of Zhu et al. [28]. The ends of the blue lines are the errors of WLS with and without ALRe.

The results are displayed in Figure 7, where the result of Zhu et al. [28] is marked as WLS+WCA (weighted constant assumption). As can be observed, naive WLS improves edge consistency but barely improves haze removal quality. With the help of ALRe, both improvements are significant and even comparable to Zhu et al. [28].

### 6.2. Disparity Refinement

Middlebury [32] is a widely used dataset with stereo images and groundtruth disparities. The ER (error ratio) of estimated disparity map is used for quality measurement. It is the percentage of bad pixels with absolute error no less than 1 (pixels with unknown disparity are excluded from statistics). As pointed out by Ma et al. [11], textureless region dominate the statistics while outliers are trivial. Therefore, we only use the 2001 dataset, where the ERs of initial results are all less than 5.

The size of WMF is 11×11 based on the configuration of Ma et al. [11]. A larger WMF could erase more outliers but also smear image contents. However, finding outliers before modifications based on a large ALRe is safe. Therefore, we set the size of ALRe to 21×21. For each sample, we produce the rough disparity map based on cost-volume method [11] (boxfilter for cost aggregation), the refined map based on WMF without ALRe and the refined map with ALRe. Disparity maps are evaluated by ESR and ER.

**Figure 8:**
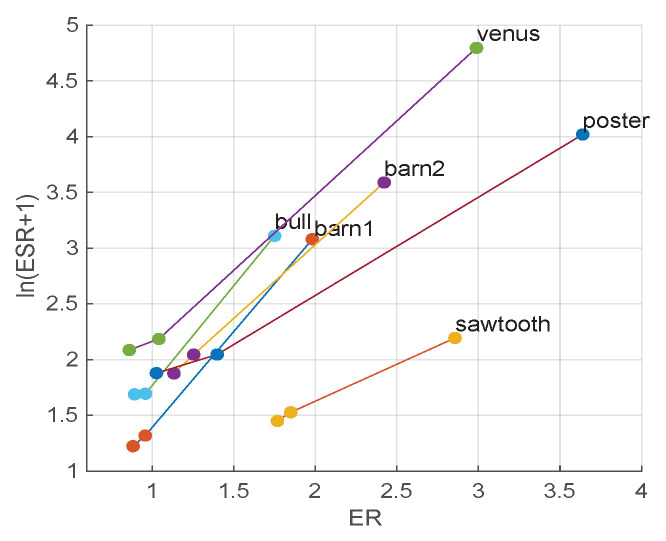
Effect of ALRe on WMF in disparity post-refinement. The errors of cost-volume method [25] are marked by the dots with sample names. Each first linked dot is the error of WMF, and the next dot is the error of WMF with ALRe.

**Figure 9:**
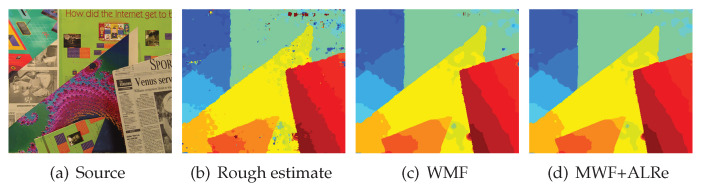
Disparity post-refinement using WMF with and without ALRe. Disparity and weight maps are displayed in color. The warmer the color, the larger the value. (**a**) One of the two stereo images; (**b**) the rough output of cost-volume [25]; (**c**) the refined result of (**b**) guided by (**a**) based on WMF without ALRe; (**d**) corresponding result with ALRe.

The results of each sample are displayed in Figure 8. The *y*-axis is ln(ESR). As can be inspected, WMF introduces significant boosts on both edge consistency and accuracy, and ALRe is able to introduce further improvements. An example of this small but stable improvement is displayed in Figure 9. As can be observed in Figure 9c,d, WMF with ALRe produces less spikes.

### 6.3. Feathering

**Figure 10:**
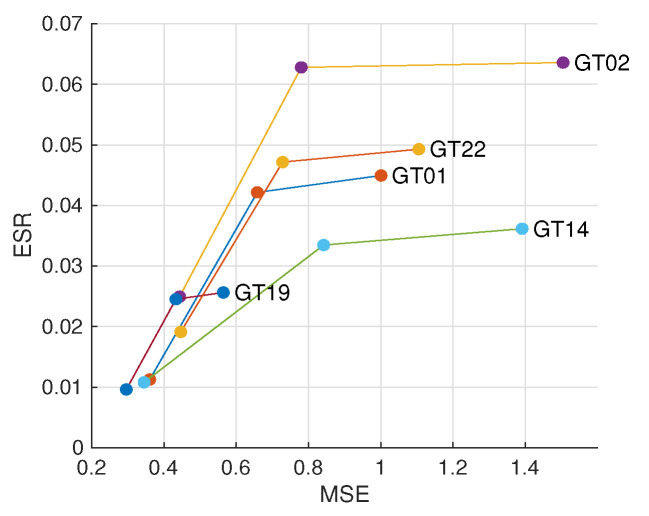
Effect of ALRe on matting. The errors of initial masks are marked by sample names. Each first linked dot is the error of GF, and the next is the error of GF with ALRe.

**Figure 11:**
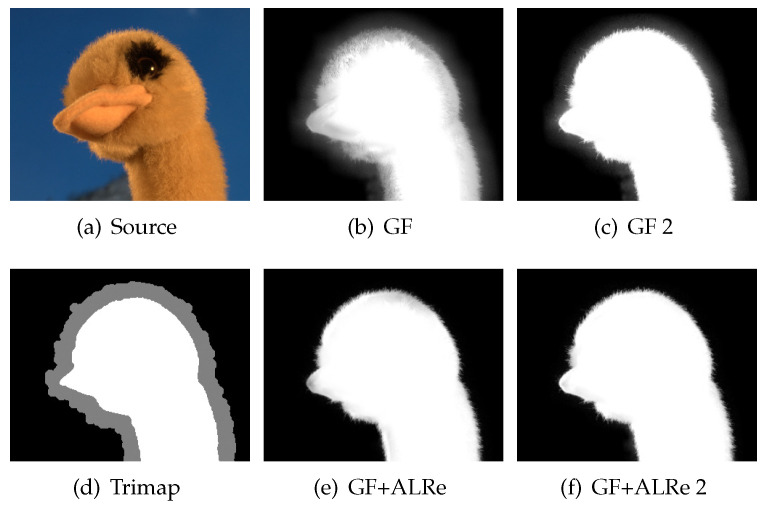
Feathering. (**a**) Color image; (**b**) the result of GF; (**c**) the result of GF with dilated foreground region; (**d**) the trimap; (**e**,**f**) corresponding results with ALRe.

Alpha Matting [33] is a dataset providing abundant images and groundtruth alpha mattes. The MSE between estimated matte and groundtruth is used for quality measurement. As shown in Figure 11d, the rough mask of Alpha matting dataset [33] is triple, including foreground region, background region and unknown pixels. To produce rough binary masks, we fill each unknown pixel by the label of its closest region. To prevent over-large errors, samples containing unknown pixels far from both regions (the Euclidian distance is more than 25) are excluded. Finally, the five samples in Figure 10 are selected.

The size of GF is 35 × 35. A larger GF could cover more reliable labels but worsen the halo-effect and texture-copy problems. However, finding outliers based on a large ALRe is safe. Therefore, we use a 51 × 51 ALRe, which can stride over the largest unknown region. For each sample, we produce the rough binary mask, the refined matte based on GF without ALRe and the refined matte with ALRe.

The results of each sample are displayed in Figure 10, where GF introduces improvements on both aspects and ALRe helps GF to introduce more. An example is illustrated in Figure 11. The result of GF shown in Figure 11b has obvious halo-effect and texture-copy problems due to its large size. However, the large size is necessary because the large unknown region. If we dilate the foreground region, the problems become less obvious but still exist, as shown in Figure 11c. As shown in Figure 11e and Figure 11f, with the help of ALRe, the problems are solved, and the refined mattes are barely affected by the dilation.

### 6.4. Outlier Detection

The IoU (intersection over union) of outlier detection result and groundtruth is used for evaluation.

(18) $$\mathrm{IoU}\left(\mathrm{GT},\mathrm{MASK}\right)=\frac{\left|\mathrm{GT}\cap \mathrm{MASK}\right|}{\left|\mathrm{GT}\cup \mathrm{MASK}\right|}.$$

MASK and GT are binary images, whose pixel *x* equals 1 if p(x) is asserted as an outlier. The MASK of ALRe is

(19) $${\mathrm{MASK}}_{\mathrm{ALRe}}{\left(p,I\right)}_{k}=\left\{\begin{array}{cc} 1,\; & \mathrm{ALRe}{\left(p,I\right)}_{k}<0.05\\ 0,\; & \mathrm{ALRe}{\left(p,I\right)}_{k}⩾0.05 \end{array}\right..$$

The MASK of WMF is produced based on the intuition that pixels greatly changed after filtering are outliers; thus,

(20) $${\mathrm{MASK}}_{\mathrm{WMF}}{\left(p,I\right)}_{k}=\left\{\begin{array}{cc} 1,\; & {\left|\mathrm{WMF}\left(p,I\right)-p\right|}_{k}>0.3\\ 0,\; & {\left|\mathrm{WMF}\left(p,I\right)-p\right|}_{k}⩽0.3 \end{array}\right..$$

Source images I are provided by Middlebury 2014 [35]. The groundtruth estimate *q* is produced based on parameter map (a,b) as $q_{k}=a_{k}^{T}I+b_{k}$. The map (a,b) is generated by smoothing a fully random matrix by R×R boxfilter. Contaminated estimate *p* is synthesized by adding outliers to *q*. Two kinds of outliers are tested, including spikes and regions. The number of outliers is controlled by *M*.

The task is more challenging with smaller *R* and larger *M*. We tested 22 kinds of *R* and 3 kinds of *M*. The size of Ω for ALRe and WMF is 25×25 (the inputs are 640×480). Larger *R* leads to larger mean(ALRe(q,I)), which indicates the fidelity of the local linear assumption. It is the cornerstone of ALRe, and therefore, we term it as ALRe expectation.

**Figure 12:**
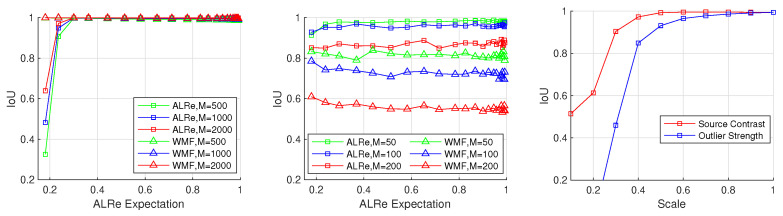
Outlier detection accuracy. Left, spike detection; Middle, bad region detection; Right, the effects of source contrast and outlier strength.

**Figure 13:**
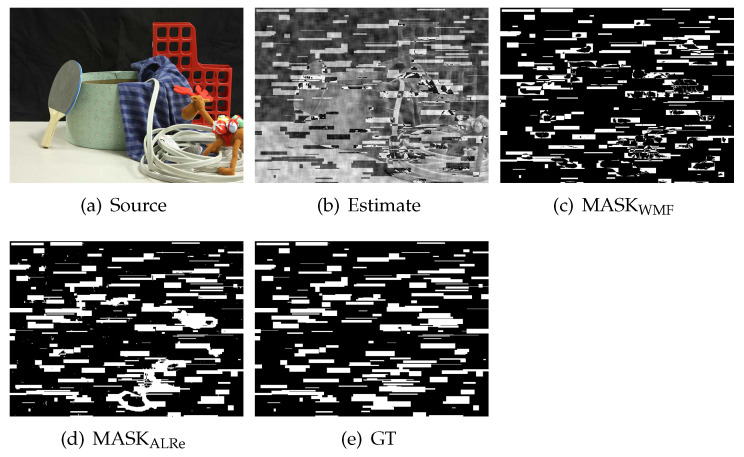
Comparison of ALRe and WMF on bad region detection. (**a**) Source image; (**b**) contaminated estimate; (**c**) detection result of WMF, IoU = 0.6508; (**d**) detection result of ALRe, IoU = 0.9027; (**e**) ground truth.

The results are illustrated in Figure 12. As can be seen, both ALRe and WMF achieve high accuracy at spike detection. ALRe fails when the ALRe expectation is lower than 0.2. The fewer the outliers, the worse the IoU. It means that ALRe has a true negative problem when the local linear assumption is not established. However, ALRe performs significantly better than WMF at bad region detection. The mean IoUs of ALRe corresponding to the increasing *M* are 0.978, 0.958, 0.868, while the ones of WMF are 0.814, 0.727, 0.556. The gap is approximately 0.2. ALRe is robust in this task even though the ALRe expectation is very low. For example, detecting outliers from Figure 13b (the ALRe expectation is less than 0.2) is very challenging. WMF only achieves an IoU equals 0.6508 because many outliers happen to be the medians. However, ALRe remains a high IoU equals 0.9027. The undesired hollows in ${\mathrm{MASK}}_{\mathrm{WMF}}$ are mostly avoided in ${\mathrm{MASK}}_{\mathrm{ALRe}}$. Furthermore, the effects of source contrast and outlier strength are tested by shrinking the intensities of *p* and I toward their mean values. As shown in Figure 12, ALRe maintains high IoUs until the contrast is lower than 70% or the outlier strength is lower than 60%.

## 7. Conclusions

In this paper, we address the problem of general outlier detection. Edge consistency is exploited, based on which, a hypothesize-and-verify algorithm termed ALRe is proposed. Analysis and comparison with other options shows that ALRe has good features including asymmetry, no false positive problem and linear complexity. Examples on four applications show that ALRe could provide meaningful detection result and even replace specifically designed method without obvious quality degradation. Experiments on three datasets demonstrate that ALRe could help post-refinement algorithms to further improve contaminated estimates. Experiments on synthetic images show that ALRe is as effective as the mainstream WMF at spike detection and is significantly better at bad region detection. The improvements measured by IoU are about 0.2.

In conclusion, ALRe is a feasible solution of the general outlier detection problem. It can be used when the estimation process is unknown, when a specific method is absent, or simply as an extra add-on of post-refinement algorithms.

## Data Availability

Publicly available datasets were analyzed in this study in Section 6.
